# Developing a Set of Core Patient‐Reported Outcomes for Kidney Replacement Therapy: A Modified Delphi Study

**DOI:** 10.1111/jorc.70069

**Published:** 2026-07-01

**Authors:** Jessica Nikolovski, Brendan Smyth, Shyamsundar Muthuramalingam, Stephen McDonald, Jessica Roydhouse, Olalekan Lee Aiyegbusi, Claudia Rutherford, Amy Luchterhand, Paolo Cardelli, Rachael L. Morton, the ANZDATA PROMs Delphi Group

**Affiliations:** ^1^ NHMRC Clinical Trials Centre University of Sydney Sydney NSW Australia; ^2^ Department of Renal Medicine St George Hospital Kogarah NSW Australia; ^3^ Barossa Hills Fleurieu Local Health Network Adelaide SA Australia; ^4^ School of Medicine Adelaide University Adelaide SA Australia; ^5^ Australia and New Zealand Dialysis and Transplant Registry Adelaide SA Australia; ^6^ Central Northern Adelaide Renal and Transplantation Service Royal Adelaide Hospital Adelaide SA Australia; ^7^ Menzies Institute for Medical Research University of Tasmania Hobart TAS Australia; ^8^ Centre for Patient Reported Outcomes Research (CPROR), Department of Applied Health Sciences, School of Health Sciences, College of Medicine and Health University of Birmingham Birmingham England UK; ^9^ Faculty of Medicine and Health, Susan Wakil School of Nursing and Midwifery, Sydney Quality of Life Office (SQOLO) University of Sydney Sydney NSW Australia; ^10^ The Daffodil Centre The University of Sydney, a Joint Venture with Cancer Council NSW Woolloomooloo NSW Australia

**Keywords:** chronic kidney failure, patient‐reported outcome measures, quality of life, registries, symptom assessment

## Abstract

**Background:**

Patient‐reported outcome measures can highlight the impact of kidney failure and kidney replacement therapy on a person's health‐related quality of life and symptom burden. But their implementation is limited and heterogeneous within Australian and New Zealand kidney care.

**Objective:**

Identify one generic health‐related quality of life and one kidney‐specific patient‐reported outcome measure suitable for inclusion within the Australia and New Zealand Dialysis and Transplant Registry, including the mode and location of administration, timing and frequency of completion, and data presentation format.

**Design:**

A three‐phase modified Delphi study comprising two online surveys and a virtual consensus workshop.

**Participants:**

Patients, carers, clinicians, researchers, and kidney registry staff from Australia, New Zealand and the United Kingdom.

**Measurements:**

Participants voted on: their preferred patient‐reported outcome measures, the mode and location of administration, timing and frequency of completion, and how they would like the data presented at the individual and aggregated levels.

**Results:**

Fifty‐three people consented to the study, 41 completed Survey #1, 43 completed Survey #2, and 30 attended the consensus workshop. The EuroQOL‐5 Dimensions‐5 Levels for generic health‐related quality of life and the Integrated Palliative care Outcome Scale‐Renal for kidney‐specific outcomes reached majority vote. Participants agreed on electronic completion at home or in the dialysis unit to allow for staff support if needed. Consensus was not reached on the frequency of administration; most voted for completion at the commencement of dialysis or transplant and 6‐monthly thereafter as a minimum.

**Conclusion:**

The EuroQOL‐5 Dimensions‐5 Levels and the Integrated Palliative care Outcome Scale‐Renal were recommended for collection by the Australia and New Zealand Dialysis and Transplant Registry. These will enable standardised, meaningful assessments of patients' health outcomes and support continuous quality improvement across kidney care services bi‐nationally.

## 1 Introduction

While kidney replacement therapy is life‐extending for people with kidney failure, health‐related quality of life is substantially reduced for people on dialysis (Lin et al. 2023). For those who receive a kidney transplant, health‐related quality of life improves but remains lower than that of the general population (Knobbe et al. 2024). The Standardised Outcomes in Nephrology initiative revealed people with kidney failure prioritise health‐related quality of life and life participation similarly to survival (Tong et al. 2017; Manera et al. 2017; Tong et al. 2016). However, these measures are not consistently included in quality indicators or outcome assessments for people receiving dialysis or a kidney transplant in Australia and New Zealand.

## 2 Literature Review

Patient‐reported outcome measures (PROMs) are questionnaires that capture a person's perspective on their symptoms and health‐related quality of life, providing a holistic view of health trajectories and treatment outcomes (U.S. Food and Drug Safety Administration 2009). Recently, there has been a growth in PROMs use to support the care of people with chronic conditions, with clinical quality registries identified as essential to expedite large‐scale collection of PROMs (Van Der Veer et al. 2021) and provide a vehicle to improve care and outcomes for people receiving kidney replacement therapy (Basch et al. 2016; Peipert and Hays 2017).

The Australia and New Zealand Dialysis and Transplant (ANZDATA) Registry is a clinical quality registry established to collect and report outcomes for all people treated with dialysis and kidney transplantation, to reduce variations in care. An external review recommended adding PROMs to the ANZDATA Registry's routine data collection (2018, unpublished). Despite these recommendations, fewer than one‐third of Australian kidney units collected PROMs as part of routine clinical care for people treated with dialysis, with increased staff workload stated as the major concern (Morton et al. 2020).

The Symptom monitoring WIth Feedback Trial (SWIFT), which recruited 2412 adults receiving haemodialysis across 92 dialysis units, demonstrated electronic PROM collection in dialysis units is feasible in Australia (Greenham et al. 2022; Morton et al. 2026). Within participating units, on average, 80% of individual patients provided PRO data (using an electronic tablet in clinic) (unpublished).

Systematic, registry‐based measurement of PROMs has the potential to improve the safety and quality of dialysis care (Van Der Veer et al. 2021). When systematically collected and linked to registry feedback mechanisms, PROMs enable benchmarking across healthcare services, identification of best practices, and support for shared decision‐making (Ruseckaite et al. 2022). Longitudinal PROMs data can enhance understanding of disease trajectories and inform clinical management, ultimately improving quality of care and patient outcomes (Ruseckaite et al. 2022). Failure to comprehensively measure and report patient outcomes is likely to lead to overlooking variations in practice, thereby missing opportunities to improve health‐related quality of life and the substantial symptom burden experienced by many people receiving kidney replacement therapy (Fletcher et al. 2022).

To address this gap, we aimed to identify one generic and one kidney‐specific PROM suitable for inclusion in the ANZDATA Registry, including the mode, location, timing and frequency of completion, as well as desired presentation formats of data at individual and aggregated levels.

## 3 Materials and Methods

This study was approved by the Central Adelaide Local Health Network Human Research Ethics Committee (Reference Number: 21036) and reported using the Recommendations for the Conducting and REporting of DElphi Studies (CREDES) checklist (Supplementary File 1) (Jünger et al. 2017).

### 3.1 Study Design

A modified Delphi method was selected to support structured consensus building across geographically dispersed stakeholder groups, including consumers, clinicians, researchers, and registry staff (Beiderbeck et al. 2021; Evangelidis et al. 2017). A standard Delphi typically begins with open‐ended exploration and relies solely on anonymous survey rounds. Our modified Delphi process involved two anonymous online surveys and a discussion (herein ‘consensus workshop’) using pre‐selected PROMs. This aimed to reduce participant burden, accelerate decision‐making, and support an applied consensus suitable for timely registry implementation. Participants were able to express their individual opinions in the first round, with opportunity to review their answers in subsequent rounds and consider others’ answers to revalidate their decisions.

### 3.2 Participants and Recruitment

Purposive and snowball recruitment strategies were used (Obilor 2023). The ANZDATA Registry has existing stakeholders (clinicians, consumers) engaged in PROMs research, including an Advisory Committee, a PROMs Working Group and a Consumer Advisory Panel comprising nine consumers trained and upskilled in research. The ANZDATA Registry General Manager distributed the study invitation to this pool of potential participants. Clinicians were invited via professional kidney care networks across Australia. To invite international participants, study investigators distributed invitations to their kidney PROMs and/or kidney quality registry networks. Participants were also encouraged to forward the invitation to other eligible colleagues and networks. The invitation email included a participant information sheet and a link to a digital consent form administered via REDCap. Eligibility is detailed in Box 1.

Box 1 **Eligibility criteria.**

Inclusion criteria:

- Aged over 18 years *and*
- Able to speak, read and write in English *and*
- A consumer, defined as:
  ◦ Having personal lived experience of being treated (past or current) with kidney dialysis
  ◦ Caregivers or close person(s) with lived experience of being a caregiver to someone treated with kidney dialysis (past or current) *and/or*
- A nurse working in kidney dialysis or transplant, including Registered Nurses, Nurse Specialists, Nurse Educators, Nurse Practitioners, Dialysis Nurses, Certified Nursing Assistants *and/or*
- Appropriately credentialed physicians (e.g., nephrologists, palliative care) with experience in kidney dialysis or transplant *and/or*
- Staff employed by the ANZDATA Registry *and/or*
- Researchers with experience in patient‐reported outcome measures in kidney conditions *and*
- Willing to engage in the Delphi process and share their views and experiences.

Exclusion criteria:

- Individuals under the age of 18; cannot provide consent and/or do not have relevant lived experience, clinical training, registry involvement, or research expertise in kidney care or patient‐reported outcome measures.

Although no formal numeric recruitment targets were set for each stakeholder group, the initial recruitment goal was approximately 30 participants. When consumer representation was initially lower, targeted recruitment through established consumer channels (e.g., consumer advisory panel meetings and professional networking platforms like LinkedIn) was undertaken, resulting in increased uptake. Our study was supported by a project support staff member who identified as an Indigenous Australian and engaged with Indigenous consumers to invite them to participate.

### 3.3 Data Collection

#### 3.3.1 Candidate PROMs

In the survey rounds, participants rated each measure individually. At the workshop, participants considered and voted on combinations of measures to identify preferred sets. The PROMs that were considered in this study were the European Quality of Life 5‐Dimension 5‐Level Version (EQ‐5D‐5L), Edmonton Symptom Assessment System Revised Renal (ESAS‐r: Renal), Integrated Palliative Outcome Score – Renal (IPOS‐ Renal), Kidney Disease Quality of Life 36‐Item Short Form Survey (KDQOL‐36), Patient‐Reported Outcomes Measurement Information System (PROMIS) Profile 29 (PROMIS‐29) and the 36‐Item Short Form Health Survey (SF‐36).

Candidate PROMs were informed by study investigators, a broad review of the published literature, and consideration of instruments used in international kidney registries. PROMs were selected based on their relevance to health‐related quality of life and symptom burden, evidence of validation and use in the target population, acceptability to patients and clinicians, and practical feasibility for large‐scale registry implementation (including length, licensing, and compatibility with existing systems).

#### 3.3.2 Survey #1 and #2 Structure and Content

Two rounds of surveys were administered via REDCap (Supplementary File 2). Participants were given 2 weeks to complete each survey, with JN sending a reminder email before the deadline. There were three sections:

1. *Demographics*. Country of birth was included as a proxy indicator of cultural and linguistic diversity, while country of residence reflected the healthcare system context in which participants were practicing or receiving care, both of which were relevant to PROM selection and implementation considerations.
2. *Usefulness ratings of six PROMs using a table comparing their characteristics*. In determining which PROM(s) progressed to Survey #2, the proportion of participants who selected the top two categories, “mostly” and “very useful”, was totalled. Participants rated the perceived usefulness of each measure on a 5‐point scale: 0 = Not at all useful, 1 = A little useful, 2 = Moderately useful, 3 = Mostly useful, and 4 = Very useful.
3. *General questions about PROMs*. Mode and location of administration, preferences for PROM data presentation.

#### 3.3.3 Criteria for Progression

The results from Survey #1 were collated and analysed. In Survey #2, participants reviewed the results of Survey #1 and had the opportunity to alter or retain their selections. For both surveys, >60% of participants selecting an item indicated agreement; 25‐60% would progress to the next round to allow participants to reconsider choices; and < 25% was considered minority opposition and would not progress to the next round (Lange et al. 2020).

#### 3.3.4 Free‐Text Analysis

Free‐text responses were analysed using descriptive content analysis. All comments were exported verbatim into Microsoft Excel and organised by participant. Responses were reviewed and coded by JN into discrete topics reflecting reasons for PROM preference (e.g. brevity, kidney specificity, coverage of psychosocial domains, feasibility for clinical use). Codes were iteratively refined as new topics emerged. The number of participants endorsing each topic was then quantified and summarised descriptively to contextualise quantitative results.

#### 3.3.5 Consensus Workshop

The consensus workshop presented results from both survey rounds, discussed each of the five decisions in detail, and used a raised‐hand vote to tally responses and determine the majority. See Supplementary File 2 for the consensus workshop agenda and topic guide. ANZDATA Registry staff presented draft data presentation formats to the core research team for initial feedback before the workshop.

The consensus workshop brought together representatives from Australia and New Zealand (international collaborators declined due to time zone differences). It lasted 2 h, was audio‐recorded, and transcribed using Zoom to assist with synthesis. Facilitators were the first author (doctoral candidate with experience in consumer advocacy) and the senior author (Professor of Health Economics, leading research about PROMs use in kidney care).

## 4 Results

Results are presented according to five decisions: (1) generic and kidney‐specific PROM to be used by the ANZDATA Registry, (2) mode of completion, (3) location of completion, (4) timing and frequency of administration, and (5) preferences for PROM data visualisation. Within each domain, findings are reported sequentially, beginning with Survey #1, followed by Survey #2, and concluding with a synthesis of the consensus workshop discussion and the final decision reached.

### 4.1 Participant Characteristics

Of the 53 participants who consented to the study, 9 withdrew, and 36 were common to both surveys (82%). Survey #2 included additional participants who had consented but not completed Survey #1, explaining the increase in participants between surveys. Survey #1 and #2 had similar participant characteristics. Most participants were aged 36–55 years, female and resided in Australia. Table 1 details participant characteristics for both surveys.

**Table 1:** Survey #1 and #2 participant characteristics.

	Survey #1 (*n* = 41)	Survey #2 (*n* = 43)
Expert	*n (%)*	*n (%)*
Patient/carer	7 (17)	8 (19)
Clinician	29 (70)	30 (70)
[ANONYMISED KIDNEY REGISTRY] staff	2 (5)	4 (9)
Researcher	4 (10)	5 (12)
Years of experience	Range	Range
Patient/carer	2.5–47	3–56
Clinician, ANZDATA Registry staff, researcher	2‐35	2–35
Age Range (years)	*n (%)*	*n (%)*
26‐35	3 (7)	3 (7)
36‐45	11 (27)	11 (26)
46‐55	15 (37)	17 (40)
56‐65	11 (27)	11 (26)
66‐75	1 (2)	1 (2)
Sex	*n (%)*	*n (%)*
Females	29 (71)	31 (72)
Males	12 (29)	12 (28)
Country of Birth	*n (%)*	*n (%)*
Australia	25 (61)	27 (63)
New Zealand	6 (15)	6 (14)
United Kingdom	—	4 (9)
France	4 (10)	1 (2)
Scotland	1 (2)	—
Colombia	1 (2)	1 (2)
Hong Kong	1 (2)	1 (2)
Saudi Arabia	1 (2)	1 (2)
Taiwan	1 (2)	1 (2)
Country of Residence	*n (%)*	*n (%)*
Australia	31 (76)	34 (79)
New Zealand	9 (22)	8 (19)
United Kingdom	1 (2)	1 (2)

Thirty participants attended the consensus workshop, comprising 18 clinicians (nephrologists, nurses, and supportive care specialists), 7 consumers with lived experience, 3 ANZDATA Registry staff, and 2 researchers. Participants represented diverse clinical settings across Australia and New Zealand, including metropolitan and regional dialysis units, transplant services, and kidney supportive care programmes.

### 4.2 Usefulness of Each PROM

**Figure 1:**
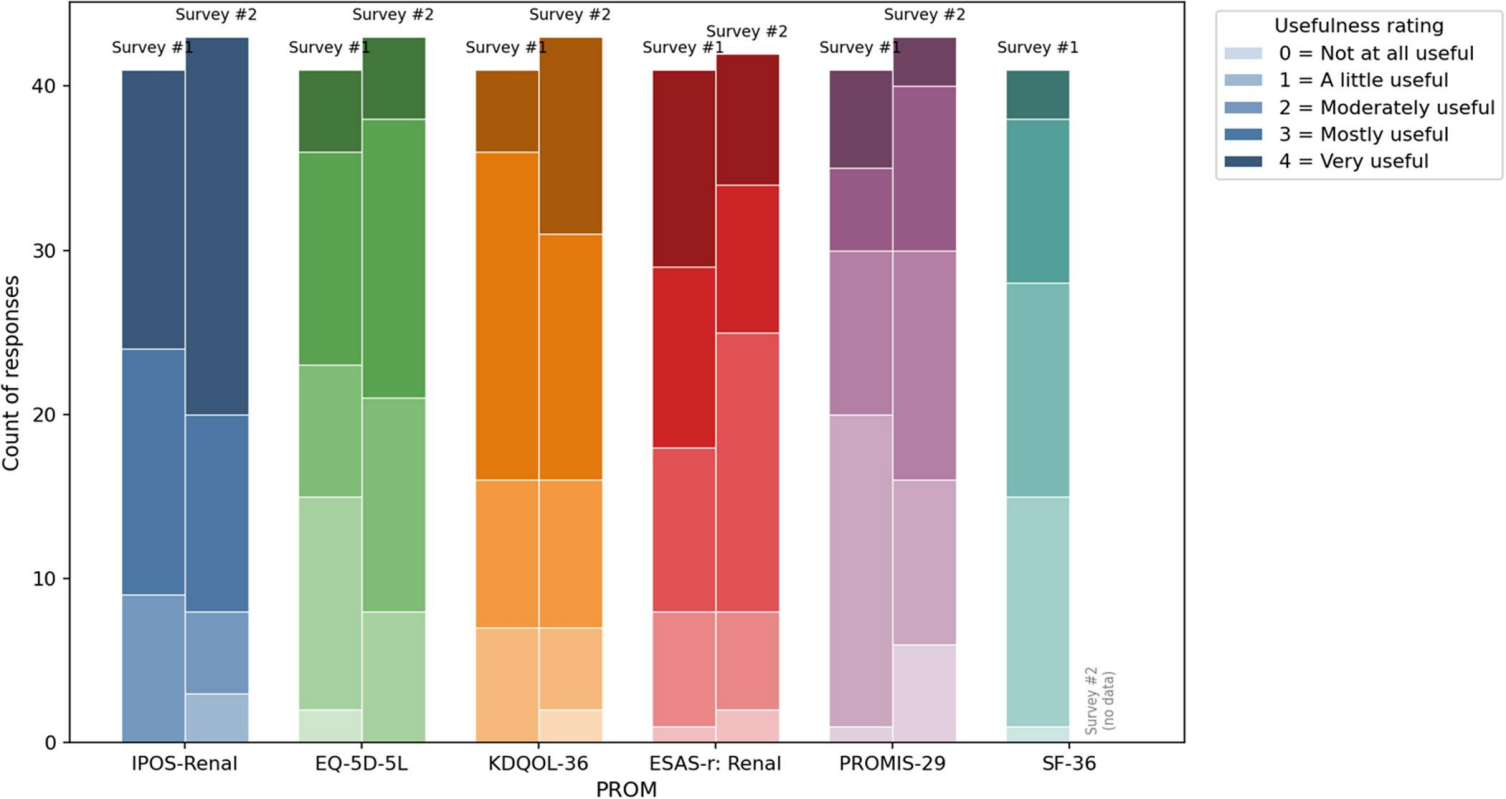
Usefulness ratings of PROMs in Survey #1 and Survey #2. Bars represent the number of respondents. EQ‐5D‐5L, EuroQoL 5‐Dimensions 5‐Levels; ESAS‐r: Renal, Edmonton Symptom Assessment System Revised Renal; IPOS‐ Renal, Integrated Palliative Outcome Score – Renal; KDQOL‐36, Kidney Disease Quality of Life 36‐Item Short Form Survey; PROM, patient‐reported outcome measure; PROMIS‐29, Patient‐Reported Outcomes Measurement Information System (PROMIS) Profile‐ 29; SF‐36, 36‐Item Short Form Health Survey.

In Survey #1 and Survey #2, the IPOS‐Renal was consistently rated most useful, followed by the KDQOL‐36 and the EQ‐5D‐5L. The SF‐36 did not progress beyond Survey #1 as fewer than 60% of participants rated it as “mostly useful” or “very useful”. See Figure 1 for a detailed breakdown of usefulness ratings.

### 4.3 Top Two Preferred PROMs

**Figure 2:**
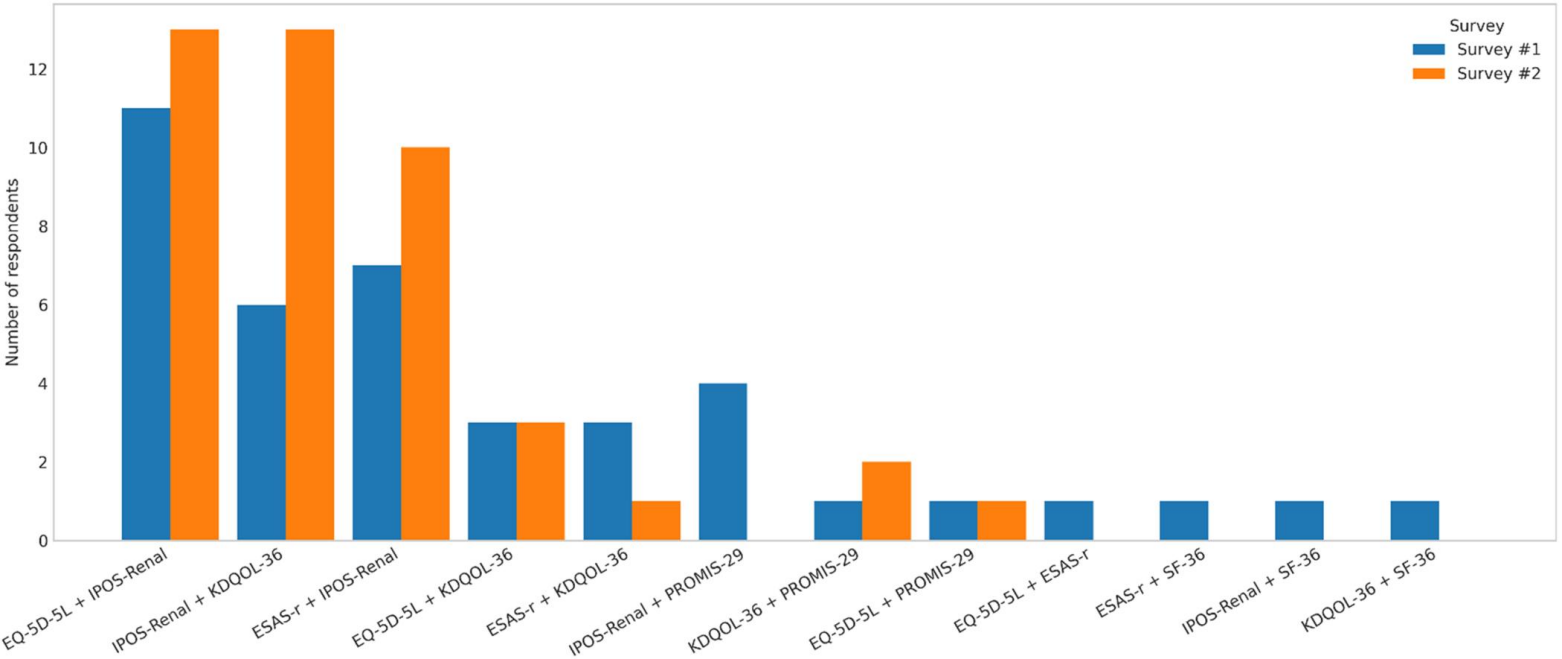
Preferred PROM combinations selected in Survey #1 and Survey #2. Bars represent the number of respondents who selected each PROM pair (rows with exactly two selections). EQ‐5D‐5L, EuroQoL 5‐Dimensions 5‐Levels; ESAS‐r: Renal, Edmonton Symptom Assessment System Revised Renal; IPOS‐ Renal, Integrated Palliative Outcome Score – Renal; KDQOL‐36, Kidney Disease Quality of Life 36‐Item Short Form Survey; PROM, patient‐reported outcome measure; PROMIS‐29, Patient‐Reported Outcomes Measurement Information System (PROMIS) Profile‐ 29; SF‐36, 36‐Item Short Form Health Survey.

After rating the usefulness of each PROM, participants were asked to select their preferred two PROMs. In Surveys #1 and #2, the EQ‐5D‐5L (generic health‐related quality of life) and the IPOS‐Renal (kidney‐specific) were the most preferred (Figure 2).

In Survey #2, participants were able to explain their top two choices using free‐text fields. Not specific to any PROM, the main reasons were: quick to complete (*n* = 15); easy to administer (*n* = 11); kidney‐specific (*n* = 18); and comprehensive coverage across different health domains (*n *= 17). Other reasons included: validation in kidney populations or widely used (*n* = 4); enabled benchmarking or comparisons for the registry (*n* = 3); multiple language options (*n* = 2); accessible for people with low literacy or poor eyesight (*n* = 2); enabled longitudinal symptom tracking (*n* = 2); enabled utility estimation (i.e., QALY weights important for economic evaluation) (*n* = 1); or possessed strong psychometric properties (*n* = 1).

In the consensus workshop, four PROMs were shortlisted based on Survey #1 and Survey #2: the IPOS‐Renal, KDQOL‐36, EQ‐5D‐5L, and ESAS‐r Renal. Before voting, participants discussed the perceived benefits and downsides of each PROM (Table 2). Several participants suggested adding a free‐text field, like the IPOS‐Renal, to all PROMs to capture issues outside predefined domains. Others proposed exploring culturally tailored measures, such as Hua Orang for Māori people (Kingi 2002; Mcclintock et al. 2013) and the Australian‐developed What Matters 2 Adults (WM2A) well‐being measure for Indigenous populations (Howard et al. 2024).

**Table 2:** Summary of participants’ feedback on each patient‐reported outcome measure (PROM) discussed in the consensus workshop.

PROM	Feedback
IPOS‐Renal	Clinicians: Symptom focus made it useful for guiding immediate care decisions, such as referrals for pain management Consumers: Lacked coverage of broader aspects of well‐being, such as the emotional impact of kidney failure
KDQOL‐36	Comprehensive: Addressed emotional well‐being, social functioning, and treatment‐related challenges, including dietary restrictions and vascular access issues Consumers: “Looks at the whole person,” including family support and financial concerns Barrier: Length and linguistic complexity for people with low literacy or from culturally and linguistically diverse backgrounds.
EQ‐5D‐5L	Advantageous for benchmarking and health economic analyses because utility scores can be generated Provided a concise snapshot of health status Researchers: Limitations in coverage of psychosocial domains beyond anxiety and depression, and potential for differing interpretation of items (e.g., people using mobility aids may report “no problems” despite functional limitations)
The ESAS‐r: Renal	Brief symptom severity scale, so easy to administer Criticised for not comprehensively covering psychosocial domains

**Final decision:** The EQ‐5D‐5L + IPOS‐Renal, noting four of seven consumers in the consensus workshop selected the KDQOL‐36 + IPOS‐Renal (Table 3).

**Table 3:** Preferred patient‐reported outcome measures in the consensus workshop (*N* = 30).

Patient‐reported outcome measure(s)	Total votes *n* (%)	Consumer votes (*n*)
IPOS‐renal + EQ‐5D‐5L	16 (62)	1
EQ‐5D‐5L + ESAS‐r: renal	0 (0)	0
KDQOL‐36 alone	0 (0)	0
KDQOL‐36 + EQ‐5D‐5L	3 (12)	2
KDQOL‐36 + IPOS‐renal	7 (27)	4
KDQOL‐36 + ESAS‐r: renal	0 (0)	0
*N*	26	7

*Note:* Only 26 of 30 participants voted.

Abbreviations: EQ‐5D‐5L: European Quality of Life 5‐Dimension 5‐Level Version; ESAS‐r: Renal: Edmonton Symptom Assessment System Revised Renal; IPOS‐ Renal: Integrated Palliative Outcome Score – Renal; KDQOL‐36: Kidney Disease Quality of Life 36‐Item Short Form Survey; PROMIS‐29: Patient‐Reported Outcomes Measurement Information System (PROMIS) Profile 29; SF‐36: 36‐Item Short Form Health Survey.

### 4.4 Preferred Mode of Completion

In all three delphi rounds, the majority preferred any option that enabled electronic completion (e.g., tablet, laptop, computer; Table 4).

**Table 4:** Preferred mode for completing PROMs (Survey #1: *n* = 41; Survey #2: *n* = 43).

Completion method	Survey #1 *n* (%)	Survey #2 *n* (%)
Any electronic option (e.g., tablet, laptop computer)	33 (81)	33 (77)
Patient's own device (e.g., mobile phone)	4 (10)	5 (12)
Tablet in hospital or clinic	3 (7)	5 (12)
Laptop/desktop computer in hospital or clinic	1 (2)	0 (0)

Participants in the consensus workshop noted that paper completion was considered a necessary fallback in the event of IT/Wi‐Fi connectivity issues or patient preference. However, concerns were raised about the cost, data‐entry burden, and delays associated with pen‐and‐paper administration. While paper completion was not preferred (or suitable) for the ANZDATA Registry, paper‐based copies could be available in clinics for patients who require them. For this approach, nurses or administrative staff could then manually upload/enter PROMs to the digital platform. Several participants recommended the registry invest in tablets, noting that electronic systems enabled real‐time feedback and longitudinal tracking and minimised burden on clinical staff (primarily nurses) to upload paper‐based PROMs into electronic data capture systems.

**Final decision:** Electronic administration.

### 4.5 Preferred Location of PROM Completion

Across all three Delphi rounds, the majority agreed that patients should have a choice of where to complete PROMs, either at home or in a hospital/clinic (Table 5). Participants in the consensus workshop thought some patients may opt to complete PROMs in their clinic if they needed staff assistance.

**Table 5:** Preferred location for completing PROMs (Survey #1: *n* = 41; Survey #2: *n* = 43).

Location option	Survey #1 *n* (%)	Survey #2 *n* (%)
Either at home or hospital/clinic	31 (76)	33 (77)
At hospital or in clinic only	8 (20)	8 (19)
At home only	2 (5)	2 (5)

**Final decision:** Patients can complete PROMs where they prefer.

### 4.6 Frequency of PROM Administration

In Survey #1, participants expressed a range of preferences for how often PROMs should be completed (see Table 6). The most frequently selected option was at the start of kidney replacement therapy, followed by every 6 months and at the time of a change in treatment modality (e.g., peritoneal dialysis to haemodialysis).

**Table 6:** Patient‐reported outcome measure administration frequency: Survey #1 (*n* = 41).

Frequency	Responses, *n* (%)
At the start of dialysis or transplantation	17 (4)
Every 6 months	15 (37)
At change of treatment type (e.g., peritoneal dialysis to haemodialysis)	11 (27)
Every 3 months	9 (22)
Every 12 months (yearly)	8 (20)
Other, please describe	4 (10)
Only when medical condition changes	3 (7)
Every clinic	3 (7)
Every 6 weeks	0 (0)
Once only	0 (0)

In Survey #2, to clarify preferences, timing options were grouped into two styles: census‐style (i.e., same timeframe for all dialysis or transplant patients) versus a personalised schedule. Responses were evenly split between these options (Table 7). For census‐style (one selection only), the preference was every 6 months. For personalised schedules (multiple selections allowed), the most frequently selected were: at the start of kidney replacement therapy; at change of treatment type; when medical condition changed. Survey #1 and Survey #2 revealed divergent views on the desired frequency of PROM completion, with no clear consensus. Therefore, participants in the consensus workshop were reminded that they were being asked to agree on a minimum requirement for the bi‐national dialysis and transplant population, and that this minimum did not preclude clinical staff from administering PROMs more frequently at their discretion.

**Table 7:** Patient‐reported outcome measure administration frequency: Survey #2 (*n* = 43).

Completion schedule	Responses *n* (%)
Census‐style (same timeframe for all patients in registry)	21 (49)
Every 3 months	4 (19)
Every 6 months	9 (43)
Every 12 months	8 (38)
Personalised schedule (per patient)	22 (51)
Start of dialysis or transplant	15 (68)
At change of treatment type (PD to HD)	14 (64)
Regular intervals	19 (86)
6 weekly	1 (5)
3 monthly	6 (32)
6 monthly	7 (37)
Yearly	4 (21)
Every clinic	1 (5)
When medical condition changes	8 (36)
Other	1 (5)
Once only	0 (0)

Clinicians and consumers requested that PROM collection align with clinical care rather than be conducted solely for registry purposes (e.g., research, quality assurance, and benchmarking). Concerns were raised about survey fatigue and reduced engagement if PROMs were not used at the point of care. Several advocated a hybrid approach with briefer PROMs (e.g., IPOS‐Renal) administered at regular intervals, and annual administration of longer PROMs such as KDQOL‐36.

Preferences for timing interacted with participants’ preferred location for administration and the purpose of completing the PROM. For example, if PROMs were used for clinical decision‐making, some consumers preferred to complete them at home to avoid feeling time‐pressured and allow for reflection. On the other hand, if patients required support (e.g., technology issues, vision impairment, translation), completing PROMs in the clinic waiting room was preferred. Some clinicians noted that if the purpose of PROM collection was benchmarking and quality assurance, census‐style completion across the entire registry would be preferred in units to reduce the burden on clinical and administrative staff.

After discussion in the workshop, three main options were considered, with the following vote:

**Option A:** Initial administration, followed by every 6 months = 14 votes.

**Option B:** Initial administration, at each change of treatment modality, and every 6 months = 6 votes.

**Option C:** Initial administration and annual follow‐up = 10 votes.

**Final decision:** Initial administration, followed by every 6 months, as a minimum.

### 4.7 PROM Data Presentation

In Survey #1 and Survey #2, consumers most frequently preferred access to their PROMs data through an ANZDATA Registry portal or website (Survey #1: 5 of 7, 71%, Survey #2: 5 of 8, 63%). Clinicians prioritised individual raw scores that were not normalised to population values in the electronic medical record for ad hoc viewing, with alerts for severe symptoms (Survey #1: 17 of 29, 59%; Survey #2: 16 of 30, 53%). In Survey #1, researchers and ANZDATA Registry staff preferred linking PROM data to electronic medical records (4 of 6, 67%), but in Survey #2, preferred aggregated hospital reports (6 of 9, 67%).

In the consensus workshop, kidney registry staff demonstrated prototype dashboards for individual and unit‐level reporting. Individual views displayed domain scores over time using line graphs, with normal thresholds and traffic‐light indicators to highlight changes. Kidney clinic‐level views used box plots to show distributions and trends. Participants agreed the visualisations were clear and clinically relevant. Some additional recommendations included allowing review of previous PROM scores, adding threshold markers to trigger clinical action, and enabling patient portal access for self‐monitoring.

**Final decision:** Patient‐level data presented as a line graph with thresholds to identify severe or worsening outcomes. Clinic‐level data presented as a box plot. Traffic light panel to highlight individual outcome trends at a glance.

## 5 Discussion

There is growing global momentum to embed the patient voice in kidney care and advance outcomes monitoring beyond traditional biomedical indicators, with PROMs as a mechanism to achieve this. The ANZDATA Registry plans to collect PROMs digitally in a standardised manner. We engaged a multidisciplinary panel to prioritise two PROMs for inclusion in the registry: the EQ‐5D‐5L (generic health‐related quality of life) and the IPOS‐Renal (kidney‐specific). Most participants voted for electronic PROM completion, where a patient prefers (home or clinic), administered at baseline and at 6‐month intervals thereafter. PROM data visualisation preferences included traffic‐light coding for stable, improving, or worsening symptoms, graphical displays with threshold markers for scores requiring clinical action, and patient access to their PROM data.

The agreed PROMs balanced clinician applicability, patient relevance, and operational feasibility for large‐scale registry use. The EQ‐5D‐5L was considered concise, enabling estimations of health utility and cross‐disease comparisons, supporting health‐economic and policy analyses. The IPOS‐Renal captured symptom burden and supportive care needs to drive improvements in kidney‐specific care and outcomes. Together, they address the multidimensional impact of kidney disease. Differences from Breckenridge et al. (2015), who shortlisted the KDQOL‐36 and SF‐12 (Breckenridge et al. 2015) may reflect evolving implementation contexts, with greater exposure to the IPOS‐Renal and EQ‐5D‐5L integrated into routine care through existing Australian state‐wide initiatives and trials such as the SWIFT.

Importantly, this Delphi process highlighted both the opportunities and challenges of implementing registry‐based PROMs. While there was broad agreement on the value of routine PROM collection, when used at the point‐of‐care to inform clinical decision‐making, concerns regarding respondent burden and additional workload for staff mirror findings from other studies (Nikolovski et al. 2026). These considerations reinforce the importance of selecting concise, validated PROMs, integrating PROMs data with electronic medical records and embedding automated feedback loops so clinicians and patients can view results that require action (Aiyegbusi et al. 2019). One clinician in the consensus workshop questioned the comparability of PROMs data across clinics with differing patient profiles (not exclusive to age, frailty, comorbidity burden, rurality, and service type). Ongoing research is exploring case‐mix adjustment methods to support valid cross‐population comparisons (Nikolovski et al. 2024a). This will be critical to ensure PROMs are interpreted appropriately and not used to draw misleading conclusions about service performance.

Our study reported consensus that PROMs should be completed electronically as the default, with paper‐based copies available for patients who require them. Evidence from Australia reported benefits of electronic PROM completion in a haemodialysis population, including the ability to enlarge text and obtain translations (Viecelli et al. 2022). People with advanced disease may require additional support to engage with electronic PROM systems, including understanding the purpose of PROMs, interpreting questions, and navigating digital interfaces (Nikolovski et al. 2026). Further, an individual's cognitive, linguistic, and functional abilities can influence their understanding, interpretation, and subsequent responses to symptom questions (Long et al. 2021). Recognising that patient trust is central to meaningful engagement, patients should be clearly informed about the purpose of PROMs, who will access their responses, and how responses will be reviewed and acted upon (Nikolovski et al. 2024b). We acknowledge this has implications for workforce capacity, requiring time and support from clinical or administrative staff. Existing mechanisms within health services, such as volunteers and dedicated registry personnel (used internationally), may help address some of the barriers related to collecting PROMs at clinical sites.

Individuals with low health or digital literacy, including those unable to read or write, may require assisted or alternative modes of completing PROMs (e.g., interviewer‐administered, proxy‐assisted, or audio‐supported formats) to ensure equitable participation and accurate data capture. Emerging approaches may also support more inclusive and efficient PROM collection in the future. The first author (JN) is conducting doctoral research to map innovative administrative methods that can reduce accessibility issues within the existing PROM infrastructure (Nikolovski et al. manuscript in preparation). These include visual or gamified features (to accommodate lower literacy and language barriers) and audio administered surveys (for people with reduced vision) (Perry et al. 2025; Soares et al. 2024). Additionally, integration with wearable devices may enable real‐time capture of patient‐reported symptoms (e.g., pruritus) (Smith et al. 2019).

Another concern is that some cultural norms may discourage disclosure of sensitive topics such as mental health or sexual wellbeing. Currently, few strategies have been reported to support PROMs uptake among culturally and linguistically diverse populations, which can result in under‐reporting or misclassification of symptoms and compromise the accuracy of patient‐reported data (Nikolovski et al. 2025). In settings like dialysis, where timely symptom recognition is critical, under‐reporting may reinforce inequities in care and outcomes, where someone does not receive appropriate or tailored care. Addressing these challenges requires culturally responsive and inclusive approaches to symptom assessment, including the use of translated and culturally‐adapted PROMs, interpreter services, and clinician training in trauma‐informed and culturally safe communication (Nikolovski et al. 2025).

### 5.1 Strengths and Limitations

As with all Delphi studies, the findings reflected the perspectives of those who participated and may not capture the full spectrum of views across all regions or care settings, despite successfully recruiting a diverse group of experts. Under‐representation of culturally and linguistically diverse participants may be due to eligibility criteria that exclude non‐English‐speaking participants. International experts in the consensus workshop were represented by participants from New Zealand; however, broader international participation was limited by time‐zone constraints. Consequently, the final consensus largely reflects Australasian perspectives. In the consensus workshop, approximately one‐third of participants had lived experience of kidney replacement therapy. While this is common in multi‐stakeholder consensus, having fewer consumers than other stakeholder groups may have influenced the final prioritised PROMs and should be noted when interpreting findings. Recruitment through existing PROM‐engaged stakeholder networks and snowball sampling may have favoured participants with prior interest in PROMs or those known to the research team or registry. Participation was voluntary, and survey responses were anonymised. Therefore, this was not considered to influence study outcomes but was important for ascertaining expert consensus.

## 6 Implications for Clinical Practice

Embedding PROMs in registry reporting systems can enhance transparency and accountability, promoting quality improvement initiatives that respond directly to the patient voice. Emerging recommendations highlight the importance of systematic PROMs integration within clinical quality registries, including guidance on measure selection, timing, and data linkage to support benchmarking and quality improvement initiatives (Ruseckaite et al. 2022). These frameworks emphasise feasibility, patient‐centred design, and interoperability with electronic health records to maximise utility for clinical care and research. Embedding PROMs within registry reporting systems could also align with existing ANZDATA Registry and Australian and New Zealand Society of Nephrology dialysis quality indicators, enabling benchmarking and unit‐level comparisons to strengthen quality assurance frameworks and support continuous improvement across dialysis services.

Although four of seven consumers in the consensus workshop preferred KDQOL‐36 alongside IPOS‐Renal, all participants in the workshop agreed the combination of the EQ‐5D‐5L and the IPOS‐Renal balanced feasibility, comprehensibility, coverage across domains and language translation. The KDQOL‐36 is a candidate for future piloting within kidney registry settings, particularly given consumer preferences from our consensus workshop and its use in the United States Renal Data System (Finkelstein 2015). Anecdotal evidence suggests other trials and clinicians have found it feasible to complete, but some items require more clarification or interpretation than those in other PROMs.

Beyond the ANZDATA Registry, registries considering PROM implementation should explore harmonisation of core measures across conditions to enable benchmarking and pooled analyses (Bull et al. 2022), invest in cultural and linguistic adaptations to ensure inclusivity (Nikolovski et al. 2025), and leverage digital platforms such as computer‐adaptive testing to reduce respondent burden (Luijten et al. 2025). Linking PROMs with clinical and administrative data, developing real‐time dashboards for clinicians, and consumer co‐design throughout implementation will help ensure the selected PROMs are relevant, understandable, and clinically useful for diverse patient populations (Ruseckaite et al. 2022; Jayasinghe et al. 2026). Ongoing evaluation of shortlisted PROMs is essential as registries refine their collection platforms and adapt to evolving patient and health system needs.

## 7 Conclusion

We identified a core set of PROMs for the ANZDATA Registry: the EQ‐5D‐5L and the IPOS‐Renal, noting four of seven consumers in the consensus workshop preferred the KDQOL‐36. Challenges such as providing resources for electronic data capture and cultural adaptation of PROMs were noted for future service planning. Embedding PROMs within the registry provides a patient‐centred approach to monitoring health status for individuals with kidney failure, guiding future treatment and referral interventions, quality improvement, and research initiatives.

## Author Contributions

**Jessica Nikolovski:** conceptualisation (lead), data curation (lead), formal analysis (lead), investigation (lead), methodology (lead), project administration (lead), visualisation (lead), writing – original draft preparation (lead), writing – review and editing. **Rachael L. Morton:** conceptualisation (lead), funding acquisition (lead), investigation (supporting), formal analysis (supporting), original draft preparation (supporting), writing – review and editing, supervision (lead). **Brendan Smyth:** conceptualisation (supporting), formal analysis (supporting), writing – review and editing, supervision. **Stephen McDonald:** funding acquisition (lead), methodology (supporting), writing – review and editing. **Shyamsundar Muthuramalingam, Jessica Roydhouse, Claudia Rutherford, Amy Luchterhand, Paolo Cardelli:** methodology (supporting), writing‐ review and editing. **Olalekan Lee Aiyegbusi:** writing – review and editing, supervision.

## 1 Conflicts of Interest

OLA receives funding from the NIHR Birmingham Biomedical Research Centre (BRC), NIHR Applied Research Collaboration (ARC), West Midlands, NIHR Blood and Transplant Research Unit (BTRU) in Precision Transplant and Cellular Therapeutics at the University of Birmingham and University Hospitals Birmingham NHS Foundation, LifeArc, Innovate UK (part of UK Research and Innovation), The Health Foundation, Gilead Sciences Ltd, Merck, Anthony Nolan, GSK, and Sarcoma UK. OLA declares personal fees from Gilead Sciences, Merck, Boehringer Ingelheim, Innovate UK, and GSK. BS declares personal fees from CSL Seqirus and AstraZeneca.

## Supporting information

Supporting File 1

Supporting File 2

## Data Availability

The data that supports the findings of this study are available in the supplementary material of this article.
