# Supplementary material for: Developing a Set of Core Patient‐Reported Outcomes for Kidney Replacement Therapy: A Modified Delphi Study
Source: J Ren Care. 2026 Jul 1;52(3):e70069. doi: 10.1111/jorc.70069 (PMC13321775; doi:10.1111/jorc.70069)
Supplement: Supplementary file 2 — Supporting File 2 [file JORC-52-0-s002.docx]

# Delphi Survey 1

Information: This survey will ask you about the usefulness of different Patient Reported Outcome Measures (PROMs) for the Australian and New Zealand Dialysis and Transplant Registry (ANZDATA); and considerations for collecting PROMs from people with kidney disease, managed with dialysis or transplant.

**After completion of all survey rounds, you will automatically be included in a PubMed listed collaborative authorship**

First you will be asked some questions about yourself before the main set of questions. Please read each question carefully.

## Part 1: Participant demographics

[*The demographic information and main question set data will be stored separately to the eConsent]*

**Please choose the best description of your role (*participants can select more than 1)*:**

1. Patient or carer with lived experience of kidney disease.
   1. *If yes, free text for number of years since diagnosis:*
2. Clinician (e.g., nurse, nephrologist)
   1. *If yes, years in profession:*
3. Professional staff (e.g., ANZDATA registry, hospital manager)
   1. *If yes, years in profession:*
4. Academic/researcher
   1. *If yes, years in profession:*

**What is your age range (in years):**

1. 18-25
2. 26-35
3. 36-45
4. 46-55
5. 56-65
6. 66-75
7. 76+

**Which of the following describes your sex?:**

1. Female
2. Male
3. Other, please describe

**How would you describe your ethnicity? (*participants can choose more than 1)*:**

1. Australian
2. Indigenous Australian or Torres Strait Islander
3. New Zealander (e.g., Māori, Pacific Peoples, NZ European, Pasifika)
4. Asian
5. Indian
6. Middle Eastern
7. European
8. African
9. Hispanic, Latino or Spanish Origin
10. Multiethnic
11. Prefer not to disclose
12. Other [please describe]:

**What country were you born in?**  *(free text: _____)*

**What country do you live in?** *(free text: _____)*

Part 2: PROM characteristics

**Please read the following table carefully. The characteristics of different PROMs are outlined below. Using this information, you will be asked to provide an overall rating of how useful each PROM is for the ANZDATA registry, when collected from patients on dialysis or with a kidney transplant.**

| **PROM** | **European Quality of Life 5 Dimensions 5 Level Version (EQ-5D-5L)** | **Integrated Palliative Outcome Score Renal (IPOS-Renal)** | **Kidney Disease Quality of Life Instrument (KDQOL-36)** | **Short Form 36 Health Survey Questionnaire (SF-36)** | **Patient-Reported Outcomes Measurement Information System® (PROMIS-29)** | **Edmonton Renal (ESAS-r:Renal)** |
| --- | --- | --- | --- | --- | --- | --- |
| Content covered | Mobility, self-care, usual activities, pain/discomfort, anxiety/depression | Symptoms (self-reported pain, shortness of breath, weakness, nausea, vomiting, poor appetite, constipation, sore mouth, drowsiness, poor mobility, itching, difficulty sleeping, restless legs, skin changes, and diarrhea), concerns, information needs | Symptoms (e.g., physical, mental, burden of kidney disease), daily life, work, social interaction, and sexual function. Generic quality of life domains in the embedded SF-12 items. | Physical functioning, bodily pain, role limitations due to physical health problems, limitations due to personal or emotional problems, emotional well-being, social functioning, energy/fatigue, general health perceptions | Physical functioning, pain interference, fatigue, sleep disturbance, social functioning, depression, anxiety, pain intensity | Pain, fatigue, nausea, depression, anxiety, drowsiness, shortness of breath, appetite, feelings of well-being, and sleep |
| General or kidney health specific | General | Kidney specific | General and kidney specific | General | General | Kidney specific |
| Number of questions | 5 | 11 | 36 | 36 | 29 | 12 |
| Validated in kidney population (Y/N) | Y | Y | Y | Y | N | Y |
| Available in electronic format (Y/N) | Y | Y | Y | Y | Y | Y |
| Available languages | Over 130 | 14 | 11 | Over 40 | Over 60 | Over 20 |
| Approximate time to complete | ~3-4 minutes | ~10 minutes | ~10-15 minutes | ~5-10 minutes | ~5 minutes | ~2 minutes |
| Is there a severity score for each topic to pinpoint specific areas of concern (Y/N) | Y | Y | Y | Y | Y | Y |
| Is there an overall score for the PROM to summarise patient’s general health? (Y/N) | Y | Y | N | N | N | Y |

**1. Please choose one option to rate the usefulness of each PROM for collection in the ANZDATA registry (This will be formatted in a matrix on REDCap):**

**EQ-5D-5L**

1. Not at all useful
2. A little useful
3. Moderately useful
4. Mostly useful
5. Very useful

**IPOS-Renal**

1. Not at all useful
2. A little useful
3. Moderately useful
4. Mostly useful
5. Very useful

**KDQOL-36**

1. Not at all useful
2. A little useful
3. Moderately useful
4. Mostly useful
5. Very useful

**SF-36**

1. Not at all useful
2. A little useful
3. Moderately useful
4. Mostly useful
5. Very useful

**PROMIS-29**

1. Not at all useful
2. A little useful
3. Moderately useful
4. Mostly useful
5. Very useful

**Edmonton Renal (ESAS-Renal)**

1. Not at all useful
2. A little useful
3. Moderately useful
4. Mostly useful
5. Very useful

**2. Overall, which two (2) PROMs would be your most preferred additions to ANZDATA:**

0, EQ-5D-5L

1, IPOS-Renal

2, KDQOL-36

3, SF-36

4, PROMIS-29

5, Edmonton Renal (ESAS-Renal)

**3. Are there other PROMs, not mentioned above, you believe should be used by ANZDATA? (*free text _____________________________________________________________________)***

***If so, why_____________________________________________________________________***

## Part 3: General PROMs questions

**4. Where do you think kidney patients should complete their PROMs?**

0, At home

1, At hospital or in clinic

2, Either at home or hospital/clinic

**5. How should the PROMs be administered?**

0, Any option that enables patients to complete PROMs electronically (e.g. tablet, laptop computer)

1, On a tablet in the hospital or clinic

2, On a laptop or computer in the hospital or clinic

3, On a patient’s own device (e.g. mobile phone, tablet, computer)

**6. How often should PROMs be administered?**

0, At the start of dialysis or transplantation

1, At change of treatment type (for example, from peritoneal dialysis to haemodialysis)

2, Every 6 weeks

3, Every 3 months

4, Every 6 months

5, Every 12 months (yearly)

6, Every clinic visit

7, Only when medical condition changes

8, Once only, for example at time of diagnosis (*free text if selected*: when should this be completed?: ________________)

9, Other (*free text ______________________________________________________*)

**7. Ideally, how should PROMs data be presented or reported back to:**

**a) Patients and carers**

0, Individualised report every 12 months

1, Individualised report every 6 months

2, Accessible through ANZDATA registry portal or website for individual patients and/or units

3, Accessible through an individual patient portal

4, Linked to patient electronic health records that they can access

5, Other (*free text ______________________________________________________*)

**b) Clinicians**

0, I do not want to see patients’ symptom results

1, Raw scores in patients’ electronic medical record (EMR) for ad hoc viewing

2, Presented as graph in EMR for ad hoc viewing

3, EMR alert only for severe or worsening symptoms

4, Accessible through ANZDATA registry portal or website for individual patients and/or units

5, In 6-monthly hospital report in aggregated form

6, Other (*free text ______________________________________________________*)

**c) Health service managers**

0, In annual hospital report in aggregated form (i.e. incorporated into standard ANZDATA report)

1, In 6-monthly hospital report in aggregated form

2, Accessible through ANZDATA registry portal or website for individual patients and/or units

3, Linked to patient electronic medica records (EMR)

4, Other (*free text ______________________________________________________*)

# Workshop

Topics for the workshop will be broad to allow for open discussion. The workshop may last between 2-3 hours and will be conducted over Zoom to allow for flexibility in scheduling participants across geographic areas and time zones.

Topic guide for consensus discussions:

- Prioritised PROMs for ANZDATA from both Delphi surveys
- Frequency of administration
- Mode of administration
- Implications and considerations for equitable access in different populations (e.g., language barriers, socioeconomic, literacy)
- Availability of support and resourcing to complete PROMs for patients unable to self-complete or versions available (e.g. large text, modified for visually impaired, language versions, etc)
